# Work, Fatigue, and Well-being: The Mediating Effect of Tiredness on Life Satisfaction in Older Adults

**DOI:** 10.1093/geroni/igaf122.2348

**Published:** 2025-12-31

**Authors:** Hyejoong Kim, Jaeon Yoo

**Affiliations:** Gachon University, Seongnam, Kyonggi-do, Republic of Korea; Gachon University, Seongnam, Kyonggi-do, Republic of Korea

## Abstract

This research examines the relationship between older adults’ labor participation and life satisfaction, with a focus on whether tiredness mediates this relationship. South Korea has the highest labor force participation rate among older adults across OECD countries, surpassing Japan (25.6%), the United States (18.9%), and the United Kingdom (10.5%). The average retirement age in South Korea is 72.3 years, which is 12.3 years higher than the statutory retirement age of 60, making it the highest among OECD nations. Even after reaching the national pension eligibility age of 63, many older adults remain in the labor market and continue to be economically active. As the elderly population grows, the number of older individuals participating in economic activities is expected to increase, making it critical to understand how work participation affects their life satisfaction. This study analyzes data from the 2019 Korea Time Use Survey, using a sample of 6,570 individuals (3,855 women (58.7%) and 2,726 men (41.5%)). The findings reveal two key insights. First, tiredness mediates the relationship between work hours and life satisfaction, meaning that longer work hours reduce life satisfaction primarily by increasing fatigue. Second, total work hours alone do not significantly impact life satisfaction, reinforcing tiredness as a key mediator. These results suggest that reducing work-related fatigue is essential for improving life satisfaction among older adults. Workplace interventions, such as flexible working hours, regular rest breaks, and fatigue management strategies, could help mitigate the negative effects of prolonged work hours and support the overall well-being of older workers.

